# Investigating the tamoxifen/high-fat diet synergy: a promising paradigm for nonalcoholic steatohepatitis induction in a rat model

**DOI:** 10.1007/s00210-024-03192-7

**Published:** 2024-06-17

**Authors:** Yousra M. Ezz-Eldin, Mohamed G. Ewees, Amany A. Azouz, Marwa M. Khalaf

**Affiliations:** 1 Department of Pharmacology and Toxicology, Faculty of Pharmacy, Nahda University, Beni-Suef, Egypt; 2 Department of Pharmacology & Toxicology, Faculty of Pharmacy, Beni-Suef University, Beni‐Suef, Egypt

**Keywords:** Tamoxifen, High-fat diet, NASH, Time point study, Ballooning

## Abstract

Non-alcoholic steatohepatitis (NASH) is a severe liver condition characterized by excessive fat deposition, ballooning, and lobular inflammation. This investigation was conducted to estimate the capability of concomitant tamoxifen administration (TAM) with a high fat diet (HFD) to induce a reliable NASH model that mimics human NASH features. Rats were administered TAM (25 mg/kg/day *p.o.*) and consumed HFD for 5 weeks. A time-course investigation was conducted to determine the optimal time for NASH development. Liver function indices, hepatic lipid profile factors, oxidative stress biomarkers, and inflammatory mediators were estimated. Additionally, macroscopic and microscopic changes were examined. Compared with the time-matched control group receiving vehicle alone, TAM/HFD significantly impaired liver function indices represented as marked elevation in ALT, AST, and ALP serum levels. TAM/HFD significantly increased lipid profile factors including high TG and TC hepatic levels. Additionally, TAM/HFD remarkably raised hepatic levels of TNF-α and IL-17 and significantly decreased IL-10. The combination also increases the oxidative status evidenced by high content of MDA as well as low activity of GPx and SOD. Accordingly, the combination of TAM and HFD for 5 weeks collaboratively promotes NASH development by initiating compromised hepatocyte functionality, elevated lipid levels, oxidative stress, and liver inflammation.

**Graphical abstract:**

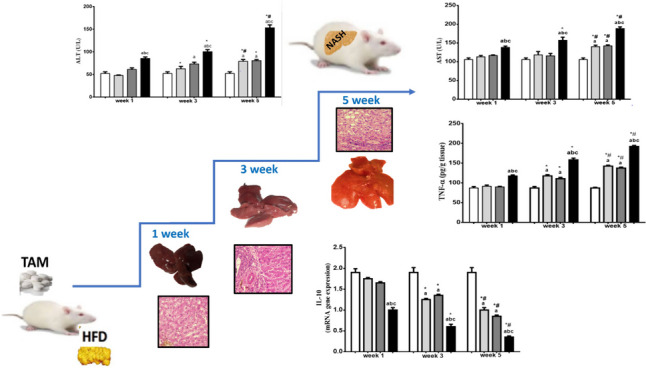

**Supplementary Information:**

The online version contains supplementary material available at 10.1007/s00210-024-03192-7.

## Introduction

Non-alcoholic steatohepatitis (NASH) represents a progressive liver condition that spans from basic lipid accumulation (steatosis) to the presence of inflammatory infiltrates, hepatocellular ballooning, and fibrosis. In the absence of alcohol consumption as a contributing factor, it can progress to more severe stages, including cirrhosis and hepatocellular carcinoma (HCC) (Zhong et al. 2020). While benign steatosis is a reversible condition that does not cause substantial impairment to liver function, prolonged exposure to steatogenic factors such as certain drugs or dietary patterns can lead to the emergence of NASH. The coexistence of steatosis and inflammation within the liver identifies this disease (Szalowska et al. 2014).

The prevalence of NASH is rising at an alarming rate. Approximately 3.6 million new cases of NASH are recorded each year. By 2030, current trends predict that there will be an estimated 101 million cases of non-alcoholic fatty liver disease (NAFLD) worldwide and 27 million cases of NASH (Estes et al. 2018).

Although NASH is often clinically silent, it is considered to be the second most frequent reason for the argued need for liver transplantation (Wong et al. 2015). Previous epidemiological studies have shown that central obesity, insulin resistance, and elevated lipid levels are substantial risk factors contributing to the development of NASH (Saklayen 2018). Approximately 82% of NASH patients are obese, 83% have hyperlipidemia, and 48% have type 2 diabetes (Younossi et al. 2016b). Additionally, different clinical studies have found a great association between NASH and the development of cardiovascular disease (Labenz et al. 2020), insulin resistance (Younossi et al. 2016a) and chronic kidney disease (Kaps et al. 2020). Based on the data mentioned above, NASH is a complex and multifactorial disease; thus, the establishment of a reliable and reproducible model that meets the clinical endpoints is a great challenge.

Directly using human models for NASH is problematic because the incidence and progression of the disease take several years and its diagnosis requires liver tissue biopsy which makes the matter restricted and inapplicable (Takahashi et al. 2012). For the fulfillment of an ideal animal model for NASH, the hepatic pathophysiological and histopathological features of human NASH should be accurately reflected to assess drug efficacy and safety properly. Accordingly, NASH manifestations should be visible in the animal liver tissue, including hepato-steatosis, intralobular inflammation, and hepatocyte ballooning. Furthermore, it should align with the following criteria concerning the progression of hyperlipidemia, insulin resistance, and inflammation (Hundertmark and Tacke 2020).

Despite numerous animal models being available, they could not fully mirror the clinical trials or accurately mimic the pathological aspects of human NASH. Therefore, it remains challenging to recognize which models most closely reflect human illness and facilitate the successful translation of research results into further clinical development (Jahn et al. 2019). Two of the most frequently used inductive models of NASH are methionine choline deficient (MCDD) diet and fat-enriched diets, which include both the western diet and the amylin diet (Flessa et al. 2022). Although these dietary models create optimal NASH histopathologic lesions, they have essential drawbacks. For instance, MCCD is uncommon among humans; very few people consume diets that are poor in methionine and choline (Rinella et al. 2008). In addition, fat-enriched diet models necessitate a longer feeding period of 25–30 weeks (Charlton et al. 2011; Clapper et al. 2013), increasing the risk of animal death from aging and other age-related issues.

Animal models of NASH are commonly created through genetic manipulation, chemical exposure, diet, or a combination thereof; however, drug-induced NASH models have not received much attention. The drug was combined with HFD because the drug alone does not produce ideal NASH (Zhong et al. 2020). Clinically, drug-induced NASH rarely occurs; only fewer than 2% of all NASH cases are triggered by drugs (Grieco et al. 2005). Additionally, our model encompasses a large category of breast cancer patients who are treated with TAM and also suffer from obesity, the matter which makes it a more realistic model.

Dietary patterns are the key strategies for controlling fatty liver diseases. High-fat diets and obesity are the main factors contributing to the global incidence of NAFLD (Abd Allah 2017). It has been shown that the liver tissue of rats subjected to a high-fat diet develops steatosis, making them more vulnerable to the chemical or pharmacological stresses that cause NASH (Anstee and Goldin 2006).

TAM is a hormonal therapy used for the treatment of breast tumors across all stages by inactivating estrogen receptors and preventing estrogen supply needed for the growth of cancer cells (Klein et al. 2013). Accumulated evidence of studies reported that TAM drives induce mitochondrial dysfunction which is considered a lipid-degrading organelle leading to micro-vesicular steatosis due to impaired β-oxidation and respiration; therefore, TAM not only induces NAFLD but also worsens fatty condition (Nishino et al. 2003; Lelliott et al. 2005; Zhao et al. 2014).

According to multiple parallel hits theory that interprets the pathophysiology of NASH, fatty liver (steatosis) is susceptible to oxidants and develops into steatohepatitis when a secondary agent, like TAM, is introduced. This secondary agent initiates liver cell death, inflammation, and stellate cell activation, potentially leading to fibrosis (Ahmed and Osman 2006). In this context, it is highly likely that concurrent treatment with TAM and HFD for chronic duration act in a synergistic manner in inducing different aspects of liver injury, so this combination should be a top priority to discuss.

In accordance with this hypothesis, obesity is considered to be a risk factor for developing breast cancer which makes the patients more vulnerable to tamoxifen-induced NASH during the therapy course (Dieterich et al. 2014). In a prospective study, obese women with high body mass index (BMI) greater than 30 kg/m^2^ were more likely to be prone to NASH than non-obese with BMI < 30 kg/m^2^ (Bruno et al. 2005). Moreover, as confirmed by Pan et al. (2016) and Saphner et al. (2009), extended use of TAM is linked to an increased risk of developing NASH, particularly among overweight women with pre-existing liver steatosis.

Consequently, the principal goal of this research was to examine the synergistic influence of TAM and HFD as an innovative strategy for inducing NASH in rats and investigate the potential molecular mechanisms involved.

## Materials and methods

### Chemicals, drugs, and kits

TAM was obtained from TECHNO Pharmaceuticals (Cairo, Egypt). Biochemical assay kits for estimation of serum alanine transferase (ALT), aspartate transferase (AST), alkaline phosphatase (ALP) and albumin were obtained from Bio-diagnostic company-Egypt (Cat. Nos. AL 10 31, AS 10 61, AP 10 20, and AB 10 10, respectively). Biochemical assay kits for estimation of tissue triglyceride (TG) and total cholesterol (TC) were obtained from BioSystems S.A. Company, Spain (CAT. Nos. 11828 and 11805, respectively). ELISA reagent kit for measurement of hepatic tumor necrosis factor-α TNF-α was purchased from Elabscience Texas-USA (CAT. Nos. E-EL-R0019). Additionally, calorimetric assay kits for measurement of superoxide dismutase (SOD) activity and glutathione peroxidase (GPx) activity as well as malondialdehyde (MDA) production in liver tissue were obtained from Bio-diagnostic company-Egypt (CAT. Nos. SD 25 21, GP 25 24, and MD 25 29, respectively). Other solvents and chemicals were of high analytical grade.

### Diet

In the present investigation, two distinct dietary regimens were employed: a regular chow diet involving 10% fat, 22% protein, and 68% carbohydrate and a HFD consisting of 45% fat, 18% protein, and 37% carbohydrate (Cheng et al. 2018).

### Animals

A total of 72 female albino rats, averaging 200 ± 30 g, were purchased from the animal facility of Nahda University (NUB), Beni-Suef, Egypt. Rats were housed in a pathogen-free environment, maintained under a 12-h dark-light cycle at optimal temperature (25 ± 1°C) and allowed free access to tape water and appropriate diet *ad libitum*.

### Work design

Rats were assigned at random into 4 main groups (*n* = 18 rats/group) as follows: (1) group I (normal control): rats fed a standard chow diet and administrated vehicle (1% tween 80 in normal saline) orally (1 ml/kg/day); (2) group II (TAM): rats fed a standard chow diet and treated with tamoxifen (25 mg/kg/day) orally; this dose was selected according to the results of our preliminary studies; (3) group III (HFD): rats fed HFD and administrated vehicle orally (1 ml/kg/day); and (4) group IV (NASH group): rats fed a HFD and treated with tamoxifen orally (25 mg/kg/day). The experiment lasted for 5 weeks, during which a time-point pattern was applied to select the optimal time for induction of NASH. Six rats from each group were euthanized after 1, 3, and 5 weeks, respectively.

### Sample preparation

After each time point, blood samples were taken in non-heparinized tubes via puncture of the eye vein, medial epicanthus, under anesthesia with diethyl ether. The blood samples were left at room temperature to clot and then immediately centrifuged at 3000 rpm and 4°C for 15 min. The supernatant was separated using a micropipette in plain sterile tubes and stored in a deep freezer at −20°C until subsequent use in assessment of biochemical parameters.

Then rats were euthanized by decapitation by well-skilled personnel. The whole liver of each rat was examined with naked eye and photographed for detection of lesions then dissected, weighed, rinsed in ice-cold isotonic saline solution, and dried out. Small specimens were taken from different lobes and kept in Davidson’s fixative solution obtained from Al-Gomhorya Company (Cairo, Egypt) for routine histopathological assessment. Another part of liver tissue was utilized to prepare the homogenate in phosphate buffer (0.1 M; pH7.4). Centrifugation was performed on this homogenate for 20 min at 2000 rpm and 4°C. The resulting supernatant was stored at −80°C until it was used to determine TC, TG, and oxidative biomarkers. Other small sections were also kept at −80°C for quantitative real-time RT-PCR analysis.

### Assessment of liver function

The evaluation of hepatic function markers, including ALT, AST, ALP, and albumin, was performed in the serum utilizing colorimetric enzymatic kits, following the guidelines provided by the manufacturer.

### Assessment of hepatic lipid profile

The researchers utilized the Bligh and Dyer method to extract lipids from various liver specimens (Bligh and Dyer 1959). The liver tissues were immersed in isopropylene, homogenized, and centrifugated at 8000 rpm and 4 °C for 5 min. The liquid portion of the mixture was subsequently gathered in order to examine the concentrations of TG and TC using commercially accessible tests, following the guidelines specified by the manufacturer.

#### Assessment of hepatic oxidative stress parameters

The supernatant collected was utilized for the biochemical analysis of GPx and SOD activity as well as MDA release. These analyses were performed using colorimetric kits in accordance with the manufacturer’s guidelines. Absorbance measurements were taken using spectrophotometric method.

### Assessment of hepatic TNF-α expression by ELISA

Expression of TNF-α in liver tissues was determined by ELISA technique following the kit procedure. The absorbance of two measurements of each sample was taken utilizing a microplate reader (ELx 800TM BioTek, USA).

### Assessment of expression of IL-10 and IL-17 genes by PCR

Qiagen tissue extraction kit (Qiagen, USA), following instructions of the manufacturer, was utilized to extract the total RNA (100 mg) from liver tissue. RNA quality and quantity were assessed using a Nanodrop spectrophotometer. High-capacity cDNA reverse transcription kit (ferments, USA) was then used to reverse-transcribe 500 ng of total RNA into cDNA according to the kit instructions. About 200 ng of cDNA was used in each PCR reaction. The following primer pair sequences were used: for **IL-10** forward: 5′- AAG GCA GTG GAG CAG GTG AA -3′; reverse: 5′- CCA GCA GAC TCA ATA CAC ACG-3′, for **IL-17** forward: 5′-GGG AAG TTG GAC CAC CAC CT-3′; reverse: 5′- TTC TCC ACC CGG AAA GTG AA-3′, and for **GAPDH** forward: 5′- GTA TTG GGC GCC TGG TCA CC -3′; reverse: 5′- CGC TCC TGG AAG ATG GTG ATG G -3′. The mRNA expression levels of IL-10 and IL-17 were measured by quantitative real-time two-step PCR performed in ABI Prism 7300 machine with ABI SYBR green system (Applied Biosystems, Forster City, CA). GAPDH parallel amplification served as the loading control. The comparative CT equation was utilized to analyze the data.

### Assessment of morphological and histopathological examinations

Liver parts hardened in Davidson’s solution were embedded in paraffin. Afterward, about 4–5-nm thickness paraffin sections were processed by conventional methods and stained with hematoxylin-eosin (H&E) dye using standard techniques previously described (Suvarna et al. 2018).

### Statistical analysis

All the data obtained from various groups were analyzed using Graph-Pad Prism-6 (Graph-Pad Software Inc., San Diego, CA). The data were expressed as the mean with standard error (mean ± SEM). A *p*-value below 0.05 was regarded as statistically significant.

## Results

### Effect of TAM/HFD combination on animal weight and relative liver weight

In this study, we observed that either TAM, HFD, or their combination affects animal’s body weight. The reduction of animals’ weight was noticed from the first week where TAM, HFD, and TAM/HFD combination decreased animal weight difference by about 79.17%, 84.38%, and 143.75%, respectively, when compared to normal control (Table 1). The reduction in animal weight increased in the third week and was markedly exaggerated in the fifth week. We noticed that TAM/HFD combination caused a marked reduction in animal weight difference by about 166.32% when compared to normal control (Table 1). The reduction in animal weight was accompanied by an increase in relative liver weight, as shown in Table 1. TAM/HFD combination induced a significant elevation in relative liver weight to about 118.64%, 127.09%, and 145.72% after 1 week, 3 weeks, and 5 weeks, respectively, compared to normal control rats (Table 1).

**Table 1:** Effect of TAM/HFD combination on animal weight and relative liver weight

	Groups	Initial wt. (g)	Final wt. (g)	Diff. in animal wt. (g)	Liver wt. (g)	Relative liver wt.
Week 1	Normal control	171.30 ± 1.25	211.30 ± 4.26	40.00 ± 5.40	6.57 ± 0.06	3.11 ± 0.06
	TAM	166.67 ± 1.67	175.00 ± 2.89	8.33 ± 3.33^a^	6.45 ± 0.21	3.69 ± 0.13^a^
	HFD	157.50 ± 4.33	163.80 ± 4.27	6.25 ± 1.25^a^	5.65 ± 0.23	3.33 ± 0.08
	TAM/HFD	173.80 ± 1.25	156.30 ± 2.39	−17.50 ± 3.22^abc^	6.20 ± 0.59	3.95 ± 0.32^ac^
Week 3	Normal control	169.10 ± 1.25	209.35 ± 4.26	39.50 ± 4.50	6.66 ± 0.04	3.10 ± 0.06
	TAM	166.30 ± 2.39	156.30 ± 4.26	−6.00 ± 0.58 ^a*^	5.65 ± 0.15	3.62 ± 0.15^a^
	HFD	158.80 ± 1.25	170.40 ± 2.04	11.25 ± 2.39^ab^	5.32 ± 0.31	3.34 ± 0.05
	TAM/HFD	173.10 ± 6.04	157.20 ± 4.63	−17.50 ± 1.44^abc^	6.20 ± 0.24	3.94 ± 0.06^ac^
Week 5	Normal control	173.50 ± 1.1	214.05 ± 3.54	49.00 ± 3.50	6.89 ± 0.05	3.04 ± 0.07
	TAM	201.25 ± 5.54	202.00 ± 4.33	−8.75 ± 2.39^a*^	7.54 ± 0.31	3.56 ± 0.06^a^
	HFD	210.83 ± 4.36	229.17 ± 3.96	18.33 ± 5.57^ab*^	7.71 ± 0.52	3.79 ± 0.04^a*#^
	TAM/HFD	210.56 ± 7.38	188.33 ± 3.22	−32.50 ± 3.35^abc*#^	7.53 ± 0.07	4.43 ± 0.11^abc*#^

Coadministration of TAM with HFD till the fifth week significantly decreased difference in animal weight as compared to the normal control group, TAM, and HFD alone as well as when compared to the first and third weeks. Each value represents the mean ± SEM (*n* = 6 rats/group). Statistical analysis was performed using two-way ANOVA followed by Tukey-Kramer multiple comparisons test where:

^a^Significantly different from Normal control group in the same week at *p* < 0.05

^b^Significantly different from TAM group in the same week at *p* < 0.05

^c^Significantly different from HFD group in the same week at *p* < 0.05

^*^Significantly different from the same group in the first week value at *p* < 0.05

^#^Significantly different from the same group in the third group value at *p* < 0.05

### Effect of TAM/HFD combination on serum liver function indices

As shown in Table 2, TAM and HFD alone revealed no significant change in the serum level of ALT, AST, ALP, and albumin when compared to those in the normal control group after 1 week. By contrast, TAM/HFD combination initiated a significant elevation in the serum level of ALT, AST, ALP compared to the normal control group, TAM group, and HFD group but with no significant change in albumin level (Table 2), whereas, after 3 weeks, a significant elevation in the serum level of ALT and ALP compared to the normal control group was noticed in rats fed HFD alone (Table 2). Meanwhile, TAM/HFD combination exhibited a significant elevation in ALT, AST, and ALP as well as a significant decline in albumin level when compared to the normal control group, TAM group, and HFD group (Table 2).

**Table 2:** Effect of TAM/HFD combination on serum levels of ALT, AST, ALP, and albumin

	Groups	Serum ALT (U/ml)	Serum AST (U/ml)	Serum ALP (U/ml)	Serum albumin (mg/dl)
Week 1	Normal control	52.25 ± 3.90	105.80 ± 4.35	314.00 ± 45.90	5.30 ± 0.21
	TAM	48.00 ± 1.15	113.00 ± 3.46	329.30 ± 17.62	5.20 ± 0.21
	HFD	61.33 ± 3.48	116.50 ± 1.76	443.70 ± 29.95	5.30 ± 0.06
	TAM/HFD	85.66 ± 3.33^abc^	138.30 ± 3.43^abc^	573.30 ± 58.69^ab^	5.08 ± 0.19
Week 3	Normal control	51.05 ± 3.21	102.50 ± 2.54	311.60 ± 45.90	5.40 ± 0.21
	TAM	62.75 ± 5.39	118.30 ± 8.99	368.20 ± 12.50	4.73 ± 0.12
	HFD	73.50 ± 3.75^a^	115.70 ± 6.36	511.30 ± 58.84^a^	4.90 ± 0.15
	TAM/HFD	100.70 ± 4.41^abc*^	157.00 ± 8.59^abc*^	747.70 ± 40.34^abc*^	4.10 ± 0.07^ac*^
Week 5	Normal control	53.36 ± 2.80	110.70 ± 3.78	316.20 ± 45.90	5.45 ± 0.21
	TAM	79.25 ± 4.31^a*#^	140.00 ± 4.04^a*#^	341.00 ± 32.84	4.43 ± 0.23^a*^
	HFD	80.60 ± 2.50^a*^	142.40 ± 2.42^a*#^	760.00 ± 33.49^ab*#^	4.60 ± 0.15^a*^
	TAM/HFD	153.50 ± 6.40^abc*#^	188.50 ± 4.48^abc*#^	1205 ± 77.60^abc*#^	3.50 ± 0.12^abc*#^

Coadministration of TAM with HFD till the fifth week significantly exaggerated serum levels of ALT, AST, and ALP and significantly decreased albumin serum levels as compared to normal control group, TAM and HFD alone as well as when compared to first and third weeks. Each value represents the mean ± SEM (*n* = 6 rats/group). Statistical analysis was performed using two-way ANOVA followed by Tukey-Kramer multiple comparisons test where:

^a^Significantly different from Normal control group in the same week at *p* < 0.05

^b^Significantly different from TAM group in the same week at *p* < 0.05

^c^Significantly different from HFD group in the same week at *p* < 0.05

^*^Significantly different from the same group in the first week value at *p* < 0.05

^#^Significantly different from the same group in the third group value at *p* < 0.05

After 5 weeks, compared to the normal control group and also compared to the third week, HFD alone significantly increased the serum levels of ALT, AST and ALP as well as significantly decreased albumin level which is similar to TAM alone (except for ALP elevation). It is noteworthy that TAM/HFD combination further exaggerated the elevation ALT, AST and ALP as well as the decrement in albumin level as compared to the normal control group, TAM group, and HFD group as well as compared to the first and third week (Table 2).

### Effect of TAM/HFD combination on hepatic lipid profile (TG and TC)

**Fig. 1:**
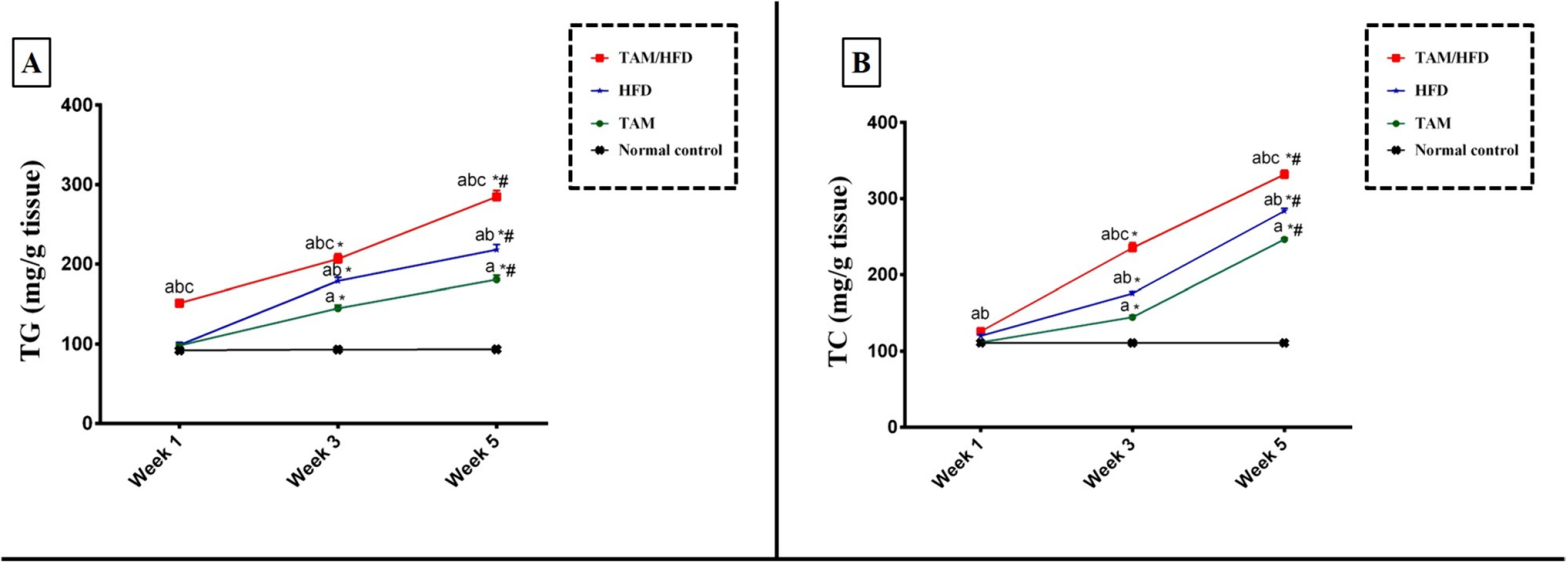
Time point study of the effect of TAM/HFD combination on hepatic lipid profile where **A**) hepatic expression of TG and **B**) hepatic expression of TC. Coadministration of TAM with HFD till the fifth week significantly exacerbated hepatic content of TG and TC as compared to normal control group, TAM and HFD alone as well as when compared to first and third weeks. Data are presented in the form of mean ± SEM (*n* = 6 rats/group). Statistical analysis was performed using two-way ANOVA followed by Tukey-Kramer multiple comparisons test. Values were considered significantly different at *P* < 0.05. ^a^vs. normal control group, ^b^vs. TAM group, ^c^vs. HFD group, ^*^vs. the same group in the first week, and ^#^vs. the same group in the third week

In this work, we observed that after 1 week, TAM or HFD alone showed no significant elevation in hepatic content of TG and TC as compared to normal control group. Contrarily, TAM/HFD combination induced a significant elevation in hepatic TG and TC contents as compared to normal control group, TAM group and HFD group (Fig. 1). After 3 weeks, the results showed that TAM and HFD alone significantly increased hepatic content of TG and TC when compared to those in normal control group whereas TAM/HFD combination promoted greater elevation in TG and TC hepatic contents as compared to the normal control group, TAM group, and HFD group as well as compared to the first week (Fig. 1).

After 5 weeks, TAM and HFD alone significantly increased TG and TC hepatic contents as compared to the normal control group and compared to the third week. Of interest, TAM/HFD combination provoked a dramatic increase in TG and TC levels as compared to the normal control group, TAM group, and HFD group as well as compared to the first and third week (Fig. 1).

### Effect of TAM/HFD combination on hepatic oxidative stress biomarkers

**Fig. 2:**
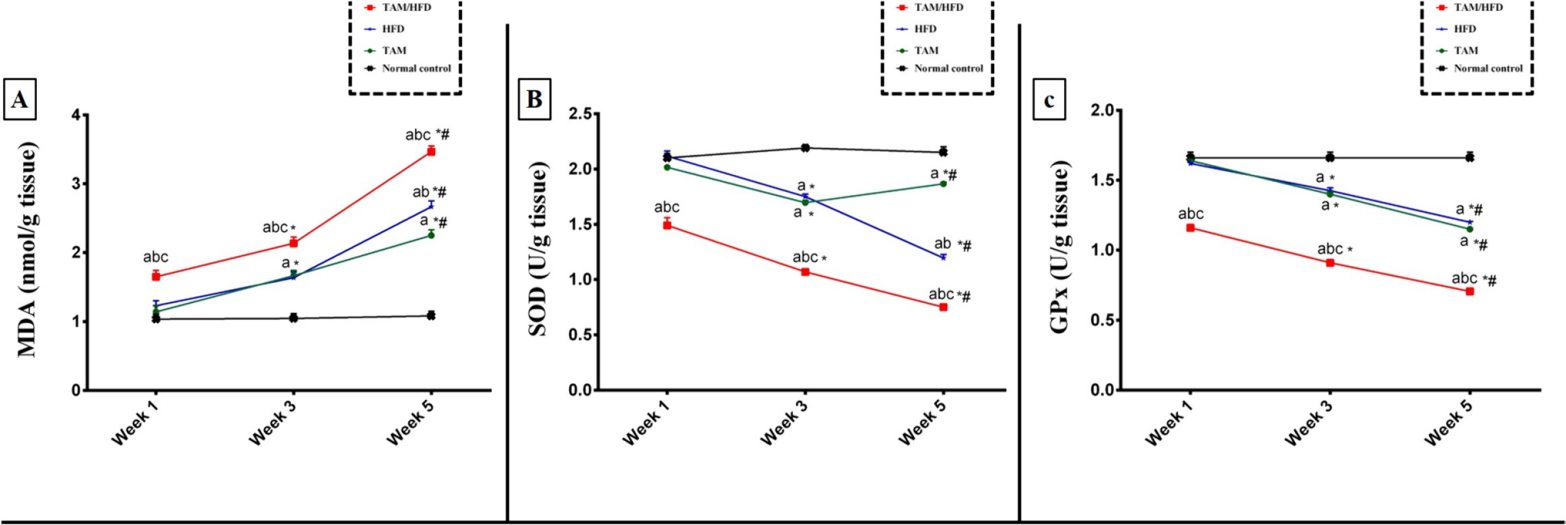
Time point study of the effect of TAM/HFD combination on hepatic oxidative stress biomarkers where **A**) hepatic content of MDA, **B**) hepatic activity of SOD, and **C**) hepatic activity of GPx. Coadministration of TAM with HFD till the fifth week significantly exacerbated hepatic content of MDA and significantly reduced GPx and SOD enzymatic activities as compared to normal control group, TAM and HFD alone as well as when compared to first and third weeks. Data are presented in the form of mean ± SEM (*n* = 6 rats/group). Statistical analysis was performed using two-way ANOVA followed by Tukey-Kramer multiple comparisons test. Values were considered significantly different at *P* < 0.05. ^a^vs. normal control group, ^b^vs. TAM group, ^c^vs. HFD group, ^*^vs. the same group in the first week, and ^#^vs. the same group in the third week

As it is shown in Fig. 2, after 1 week, no significant differences in hepatic MDA content, GPx, and SOD activities were observed in rats treated with either TAM or HFD alone compared to the normal control group. Conversely, TAM/HFD combination produces an observable elevation in hepatic MDA content and a significant drop in hepatic GPx and SOD activities as compared to the normal control group, TAM group, and HFD group (Fig. 2). We also noticed that rats treated with TAM or HFD alone revealed a significant elevation in hepatic MDA content and notable decrement in GPx and SOD activities as compared to the normal control group after 3 weeks, while rats treated with TAM/HFD combination had marked elevation in hepatic MDA release and a significant reduction in activities of GPx and SOD as compared to the normal control group, TAM group, and HFD group (Fig. 2).

After 5 weeks, TAM and HFD alone significantly increased MDA content and decreased GPx and SOD activities as compared to the normal control group. Meanwhile, TAM/HFD combination provokes a remarkable elevation in MDA content and a substantial decrease in GPx and SOD activities as compared to the normal control group, TAM group, and HFD group as well as compared to the first and third weeks (Fig. 2).

### Effect of TAM/HFD combination on expression of TNF-α protein as well as IL-17 and IL-10 gene expression

**Fig. 3:**
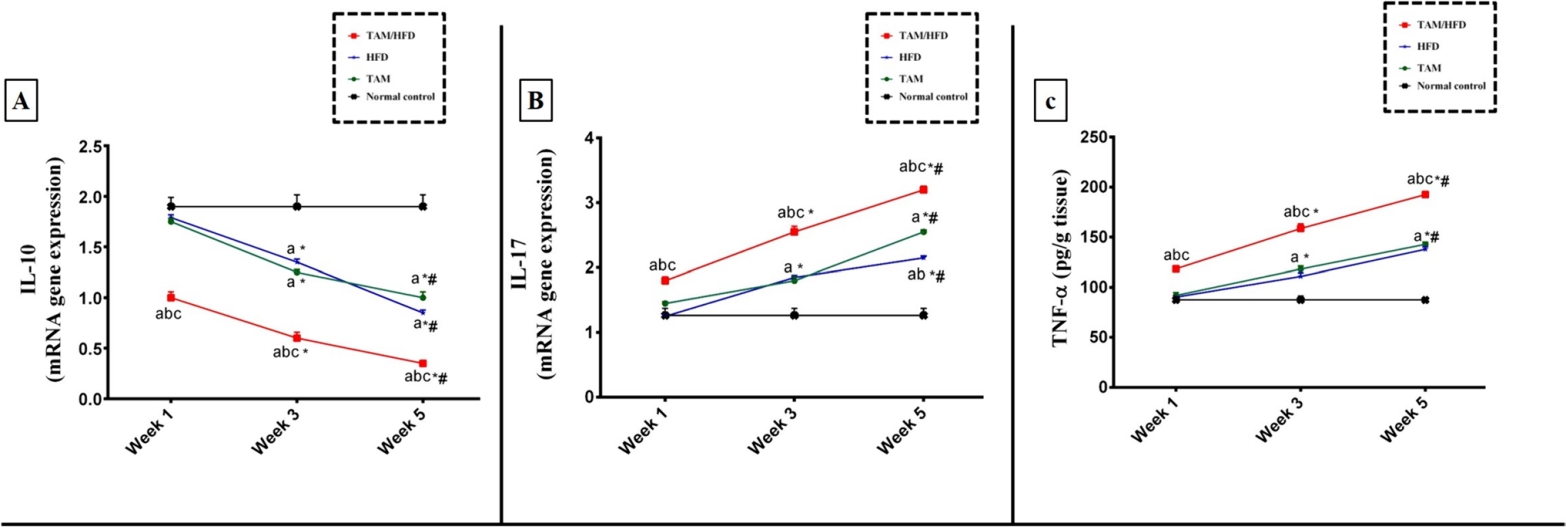
Time point study of the effect of TAM/HFD combination on expression of TNF-α protein as well as IL-17 and IL-10 gene expression where **A**) hepatic expression of mRNA gene expression of IL-10, **B**) mRNA gene expression of IL-17, and **C**) hepatic expression of TNF-α. Coadministration of TAM with HFD till the fifth week significantly reduced IL-10 as well as markedly increased IL-17 and TNF-α hepatic expression when compared to first and third weeks. Data are presented in the form of mean ± SEM (*n* = 6 rats/group). Statistical analysis was performed using two-way ANOVA followed by Tukey-Kramer multiple comparisons test. Values were considered significantly different at *P* < 0.05. ^a^vs normal control group, ^b^vs TAM group, ^c^vs HFD group, ^*^vs the same group in the first week, and ^#^ vs the same group in the third week

In our study, no significant changes were observed in the expression of TNF-α protein as well as IL-17 and IL-10 gene expression as compared to the normal control group, TAM group, or HFD group after 1 week (Fig. 3). On the other side, TAM/HFD combination produces a significant increase in TNF-α expression and IL-17 gene expression as well as a significant drop in gene expression of IL-10 as compared to the normal control group, TAM group, and HFD group (Fig. 3). After 3 weeks, TNF-α expression and IL-17 gene expression were considerably upregulated, and IL-10 gene expression was markedly downregulated in rats treated with TAM or HFD alone when compared to those in the normal control group beside comparison to the first week results, while TNF-α and IL-17 exhibited greater expression and IL-10 gene expression reached a more significant reduction in rats treated with TAM/HFD combination as compared to the normal control group, TAM group, and HFD group besides compared to the first and third weeks (Fig. 3).

Surprisingly, rats treated with TAM/HFD combination showed overexpression in TNF-α protein and IL-17 gene with considerable reduction in IL-10 gene expression as compared to normal control group, TAM group, and HFD group besides compared to the first and third week (Fig. 3).

### Effect of TAM/HFD combination on morphological and histopathological changes

**Fig. 4:**
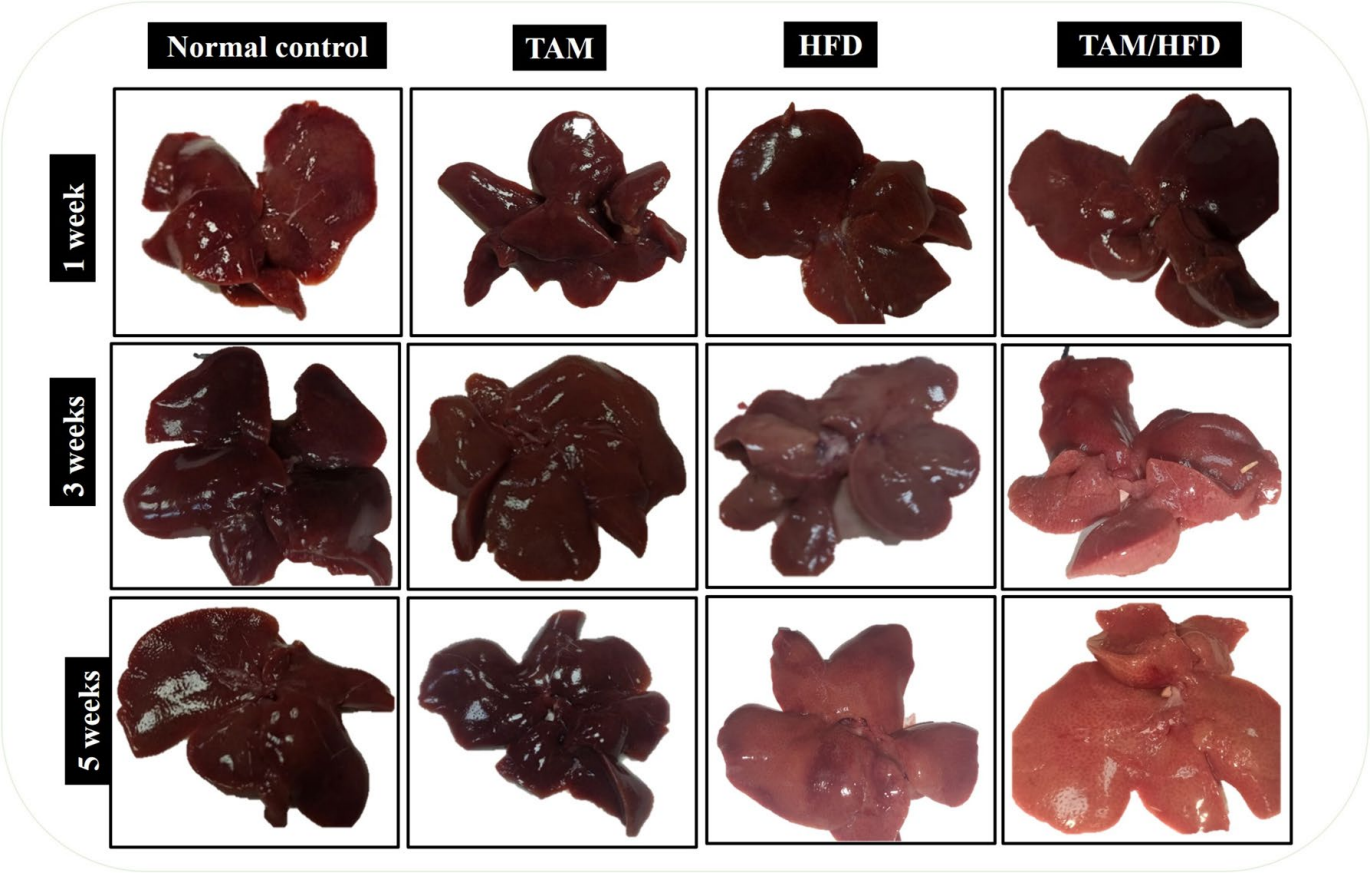
Time point study of the effect of TAM/HFD combination on entire liver morphology. Photomicrographs of the entire liver after 5 weeks demonstrated that liver tissue from the normal control and TAM groups showed reddish brown color, the HFD group showed light brown color, and coadministration of TAM with HFD showed obvious pale buff color

From week 1 to week 5, the external features of representative gross liver tissues of TAM groups exhibited normal surface touch and color, resembling the normal control group, while the HFD group exhibited gradual changes from reddish brown to yellowish brown color with increased liver size, whereas the TAM//HFD combination group exhibited progressive alternation from reddish brown to pale buff color and the tissues became easily torn (Fig. 4).

**Fig. 5:**
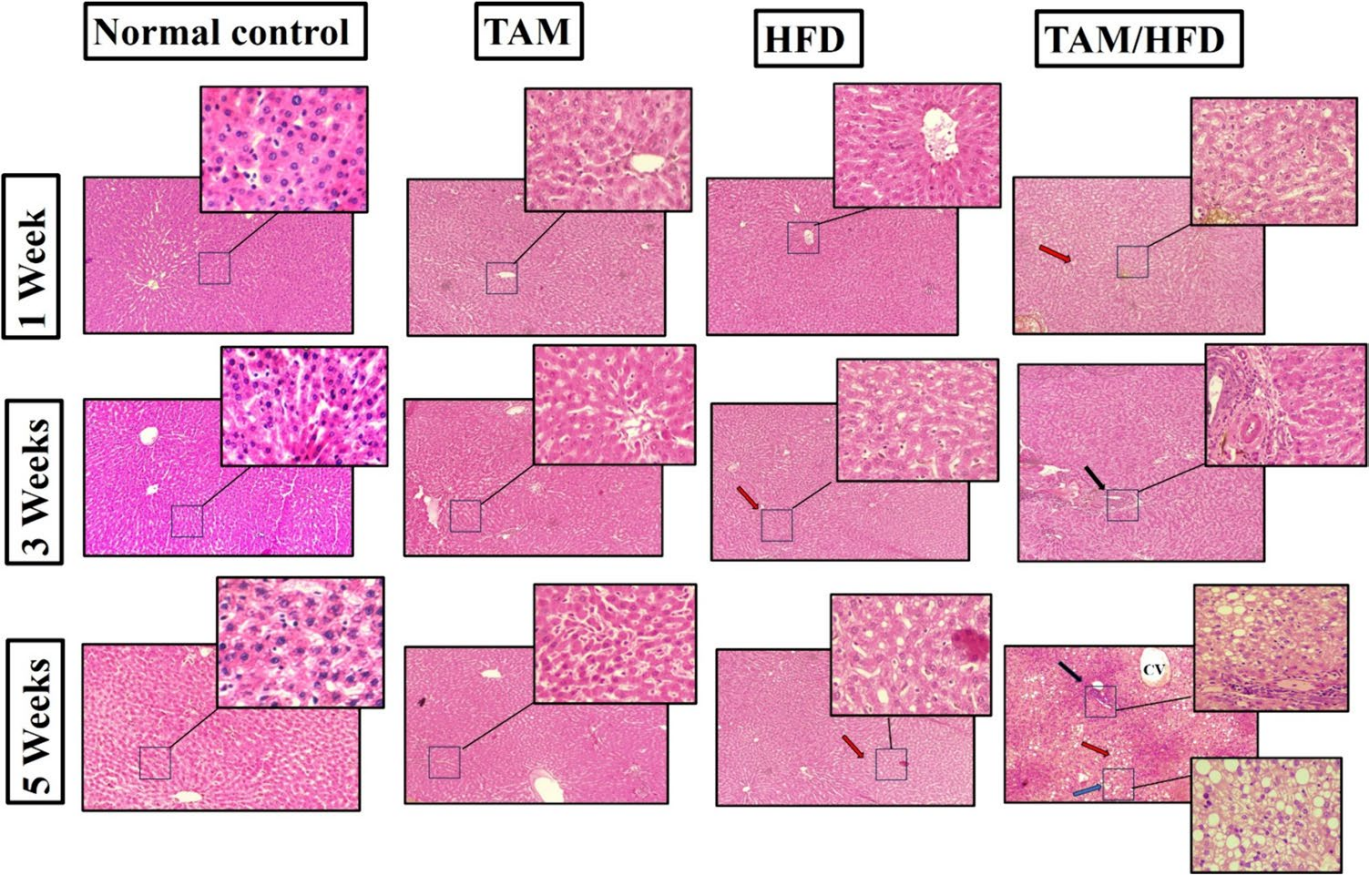
Time point study of the effect of TAM/HFD combination on histopathological changes in hepatic liver of rats treated with TAM or HFD or TAM/HFD combination; steatosis (red arrow), inflammatory infiltrates (black arrow), and ballooning (blue arrow)

The liver tissues were further analyzed histologically using H&E staining. As expected, sections taken from rats after the first week showed no histological changes across all groups. However, after 3 weeks TAM or HFD groups showed modest hepato-steatosis and ballooning along with simple inflammation. These became more apparent in the TAM/HFD group than the HFD group. These characteristics are aggravated by 5 weeks (Fig. 5).

## Discussion

The lack of valid integrated animal models for NASH stands as an obstacle to preclinical studies for novel therapeutic agents. While prior research efforts have attempted to induce liver lipid accumulation (Li et al. 2022), our study represents the first attempt to use a combination of TAM and HFD to efficiently replicate key human NASH characteristics within a short timeframe and cost-effectively with a minimized duration of malnutrition.

The disruptive effect of either TAM or HFD on mitochondrial β-oxidation, inducing liver impairment, was previously reported (Larosche et al. 2007; Vial et al. 2011; Mahmouda et al. 2021). However, our investigation was pioneering in observing the effect of the concomitant administration of tamoxifen with HFD on inducing and aggravating NASH in rats. Thus, we attempt to discuss and interpret this detrimental consequence of the combination.

On the level of liver biochemical function, we observed that administration of either TAM or HFD individually did not cause any significant alteration in the liver indices during the first and third weeks except a minimal elevation in ALT and ALP levels induced by HFD only indicating that there is insufficient time for the liver to be impaired. After the fifth week of the study, we noticed that TAM or HFD alone induced a marked elevation in serum ALT, AST, and ALP with a concurrent decrease in albumin synthesis compared to normal control. In consistency with our results, previous studies exhibited impaired liver function and hepatotoxicity after exposure to a high-fat diet regimen or TAM (Martín-Pozuelo et al. 2019; Famurewa et al. 2020).

On the other hand, we noticed that TAM/HFD cotreatment induced a significant impairment of liver function starting from week 1. Liver functions were worsening gradually by the third week, and this was exaggerated dramatically by the fifth week compared to either normal control rats or TAM as well as HFD alone. This was attributed to the high affinity of TAM to liver tissues, causing conformational changes in the cell membrane and facilitating the leakage of enzymes outside the cell (Ibrahim et al. 2014). This was evidenced by substantial exaggeration in the elevation of serum ALT, AST and ALP levels and the drop in albumin level which is the most abundant plasma protein produced by liver cells and its decrease refers to disturbance in the liver synthetic capability (Ruot et al. 2000; Famurewa et al. 2020).

It is well-established that the liver serves as the central regulator of lipid equilibrium in the body. In typical conditions, the liver processes a substantial quantity of fatty acids (FAs) while storing only a minute portion in the form of TG. This equilibrium results from the balance between the liver’s acquisition of FAs through plasma uptake and de novo synthesis, as well as the release of FAs into the bloodstream as triglyceride-enriched very low-density lipoprotein (VLDL-TG) after undergoing oxidation. The limited quantity of triglycerides stored in the liver is typically found within cytoplasmic lipid droplets. Consequently, any impairment in liver function disrupts lipid homeostasis, leading to the accumulation of TG and TC in hepatic cells (Alves-Bezerra and Cohen 2017).

In this context, the elevation in TG and TC wasn`t detectable following 1 week of induction with TAM or HFD alone since the duration is not enough to prompt dysregulation in lipid metabolism. However, after 3 weeks, administration of either TAM or HFD alone initiates slight elevation in hepatic TG and TC contents which becomes more significant by week 5 compared to normal control. These came in consistent with prior studies which return TG-elevating action to fatty acid beta-oxidation inhibited through HFD consumption or TAM administration (Cole et al. 2010; Wang et al. 2021).

Otherwise, TAM/HFD cotreatment induces an obvious increase in lipid accumulation as evidenced by significantly higher levels of hepatic TG and TC from week 1 and progressed markedly after 3 weeks. The elevation in hepatic TG and TC levels were excessively amplified after 5 weeks of TAM/HFD cotreatment compared to HFD or TAM alone. This may be explained by the ability of TAM to provoke hyperlipidemia through inhibiting mitochondrial β-oxidation leading to regression in the removal of fat from the liver (Larosche et al. 2007).

Another likely explanation of the worsening in lipid profile (TG and TC) by TAM + HFD is the inhibition of Peroxisome proliferator-activated receptor-alpha (PPAR-α) which has a pivotal function in elevating LDL and lowering HDL levels contributing to NASH aggravation (Contreras et al. 2013). Therefore, the effect of this combination on PPAR-α expression needs to be investigated.

It is noteworthy that TAM/HFD rats exhibited a reduction in body weight difference that was exacerbated by the end of week five. Evidence attributed this weight reduction in NASH group to TAM-induced ROS generation which is closely related to stimulating adipocyte dedifferentiation and upregulating apoptotic and autophagic genes that are crucial for adiposity regulation (Liu et al. 2015). Moreover, the expression of proinflammatory marker TNFα was stated to promote adipocyte dedifferentiation through downregulation of PPARγ (Xing et al. 1997). TAM administration elevated TNFα expression as mentioned above in our results.

Oxidative stress and lipid peroxidation are the principal candidates for a second hit (Lettéron et al. 1996; Day and James 1998). The excessive storage of lipids in the hepatocytes encourages the release of high levels of reactive oxygen species (ROS) and subsequent lipid peroxidation. The oxidation process of unsaturated fatty acids (FAs) or any other lipids results in the formation of peroxides of these molecules. Increased or abnormal peroxidation occurs in both hepatic cells and circulating lipids, resulting in induction of lipotoxicity and metabolic dysfunctions (Almeda-Valdes et al. 2015; Marra and Svegliati-Baroni 2018).

Moreover, the disturbance in antioxidant homeostasis caused a reduction of the hepatic activities of ROS-scavenging enzymes (SOD and GPx). This downregulation of ROS-scavenging enzymes, along with increased hepatic MDA content, a biomarker of LPO, precipitates oxidative hepatotoxicity (Lettéron et al. 1996).

In our study, we observed that after 1 week, there were no significant changes in the hepatic GPx and SOD activities as well as MDA production over the tested groups (except for the TAM/HFD combination). However, after 3 weeks, both TAM and HFD induced minimal elevation in MDA as well as decline in GPx and SOD were observed. This is compatible with the previous study of El-Kashef and El-Sheakh (2019) who exhibited that administration of TAM at a dose of 45 mg/kg for a short duration caused mild oxidative damage. Additionally, our results confirmed the results of another previous study which indicated that enzymatic antioxidants were depleted in a rat model of obesity induced by HFD (Hsu and Yen 2007).

TAM/HFD cotreatment considerably increased MDA and decreased GPx and SOD. This oxidative damage was not comparable with the damage that occurred after 5 weeks when TAM/HFD cotreatment exhibited the maximum rate of lipid peroxidation evidenced by increased MDA content as well as provoked a more dramatic decrease in GPx and SOD activities suggesting a synergizing effect of TAM with HFD in disrupting the oxidative status. Our observations are supported by a prior study that exhibited the deleterious impact of TAM on mitochondrial respiration causing generation of ROS, cytokine cascade release, and development of steatohepatitis lesions (Parvez et al. 2008).

Additionally, this observation is confirmed by previous data demonstrating that the preexisting steatotic liver is vulnerable to generation of oxidative changes when exposed to certain drugs such as TAM (Bruno et al. 2005). Additionally, our results are in agreement with other animal and human models which demonstrated that NASH disease is strongly associated with oxidative stress and abnormal peroxidation of lipids which encourages lipotoxicity (Lettéron et al. 1996; Martín-Fernández et al. 2022).

Oxidative signaling is a major trigger of proinflammatory cascades through regulation of different gene expressions where oxidative stress-driven inflammation is strongly implicated in the NASH model (Rolo et al. 2012). TNF-α is a proinflammatory mediator that has pleiotropic effects on multiple biological processes like cell apoptosis, growth, oncogenesis, and immune activity (Rashid et al. 2013). The role of TNF-α in the transition of steatosis to NASH is widely reviewed in many literatures (Tomita et al. 2006; Lu et al. 2022). Furthermore, TNF-α has been shown to decrease mitochondrial respiration and activate the mitochondrial permeability transition pore, both leading to mitochondrial dysfunction. This might cause lipid peroxidation and production of ROS in the mitochondria (Pessayre et al. 2001). When TNF-α is released, it activates redox-sensitive kinases, which can promote proinflammatory pathways and insulin resistance (Armutcu et al. 2005).

In addition, a previous study demonstrated that IL-17 has been implicated in multiple liver diseases and has a central role in driving inflammation in NAFLD model (Giles et al. 2015). Notably, IL-17 activation is recognized to increase fat uptake inside liver cells and the subsequent production of cytokines cascade (Tang et al. 2011). On the other side, IL-10, a Th2 anti-inflammatory cytokine, was reported to be downregulated in a mouse model of a diet-induced NASH (Li et al. 2021).

In our study, we noted that administration of TAM or HFD alone to rats did not result in significant changes in these proinflammatory and anti-inflammatory cytokines after 1 week, while after 3 weeks, they caused an increase in IL-17 and TNF-α and a decrease in IL-10 to a certain extent. These changes became more pronounced after 5 weeks. Matched with these findings, multiple researchers demonstrated that TAM administration raised hepatic TNF-α expression with a concurrent decrease in IL-10 expression mediating acute and chronic liver injury (El-Dessouki et al. 2018; Martín-Pozuelo et al. 2019). Similarly, a fat-enriched diet was shown to increase hepatic TNF-α and IL-17 expression worsening liver function, and causing hepatic lipid accumulation and inflammation (Lestari et al. 2021).

On the other hand, cotreatment with TAM/HFD significantly caused a further increase in the inflammatory pattern where IL-17 gene expression and TNF-α expression were remarkably overexpressed and IL-10 gene expression was remarkably downexpressed from the first week. This effect of cotreatment with TAM/HFD on inflammatory pattern was exacerbated after 3 weeks and augmented in the fifth week compared to HFD or TAM group. This may be ascribed to the upregulation of nuclear factor kappa-light-chain-enhancer of activated B cells (NF-ĸB), whose activation is responsible for the release of multiple proinflammatory mediators including IL-17 and TNF-α (Rashid et al. 2013; Park et al. 2014).

All these biochemical and molecular findings were confirmed by morphological and histological examination. According to the morphological analysis, by the end of the experiment, rats fed a fat-enriched diet showed an enlarged pale liver which indicates moderate fat accumulation. The color became paler with greater liver size in TAM/HFD which excessively promotes fat infiltration and severe hepatic lesions.

As expected, the histopathological alternations were not observed after 1 week. But with duration, HFD alone caused simple hepato-macro and microvesicular steatosis which came in consistent with Yang et al. (2021). TAM/HFD coadministration exhibited severe hepato-steatosis in parallel with ballooning degeneration and inflammation. This is meant to increase the susceptibility of this steatotic liver to more robust inflammation and ballooning degeneration. These histopathological alternations peaked by week 5.

## Conclusion

Collectively, our biochemical and molecular parameters as well as morphological and histological examinations reflect the clinical picture of NASH in rat model and provide valuable insights into the application of the TAM/HFD model as a novel approach to studying NASH in animals. Additionally, our research identifies the optimal time for observing the manifestation of key characteristics associated with NASH.

## Electronic supplementary material

Below is the link to the electronic supplementary material.

Supplementary Material 1

Supplementary Material 2

## Data Availability

No datasets were generated or analyzed during the current study.
