# Supplementary material for: Investigating the tamoxifen/high-fat diet synergy: a promising paradigm for nonalcoholic steatohepatitis induction in a rat model
Source: Naunyn Schmiedebergs Arch Pharmacol. 2024 Jun 17;397(11):9067–79. doi: 10.1007/s00210-024-03192-7 (PMC11522070; doi:10.1007/s00210-024-03192-7)
Supplement: Supplementary file 1 — Supplementary Material 1 [file 210_2024_3192_MOESM1_ESM.docx]

Table S1: Preliminary study of the effect of different doses of TAM on liver function test (ALT and AST levels) in serum.

|  | ALT  (U/L) | AST  (U/L) |
| --- | --- | --- |
| Normal Control | 55.05 ± 2.56 | 105.33 ± 1.6 |
| TAM/HFD (dose 40 mg/kg) | 270.89^a^ ± 2.6 | 350.17^a^ ± 3.1 |
| TAM/HFD (dose 30 mg/kg) | 210.45^a^ ± 4.1 | 275.24^a^ ± 3.4 |
| TAM/HFD (dose 25 mg/kg) | 112.5^a^ ± 2.4 | 168.25^a^ ± 4.1 |
| TAM/HFD (dose 20 mg/kg) | 60.5 ± 1.9 | 111.25^a^ ± 2.7 |

**Note:** in this preliminary study, different four doses of TAM 40, 30, 25 and 20 mg/kg, respectively were used. We noticed that rats treated with either 30 or 40 mg/kg showed a high elevation in liver function tests, severe body weight loss and high mortality rate the first 2 weeks of administration. On the contrary, rats injected with TAM 20 mg/kg showed no significant changes in ALT and AST levels. Meanwhile, animals treated with TAM 25 mg/kg because showed a significant elevation in AST and ALT levels with no mortality rate and therefore, this dose was selected.
