# Supplementary figures and images for: Investigating the tamoxifen/high-fat diet synergy: a promising paradigm for nonalcoholic steatohepatitis induction in a rat model
Source: Naunyn Schmiedebergs Arch Pharmacol. 2024 Jun 17;397(11):9067–79. doi: 10.1007/s00210-024-03192-7 (PMC11522070; doi:10.1007/s00210-024-03192-7)

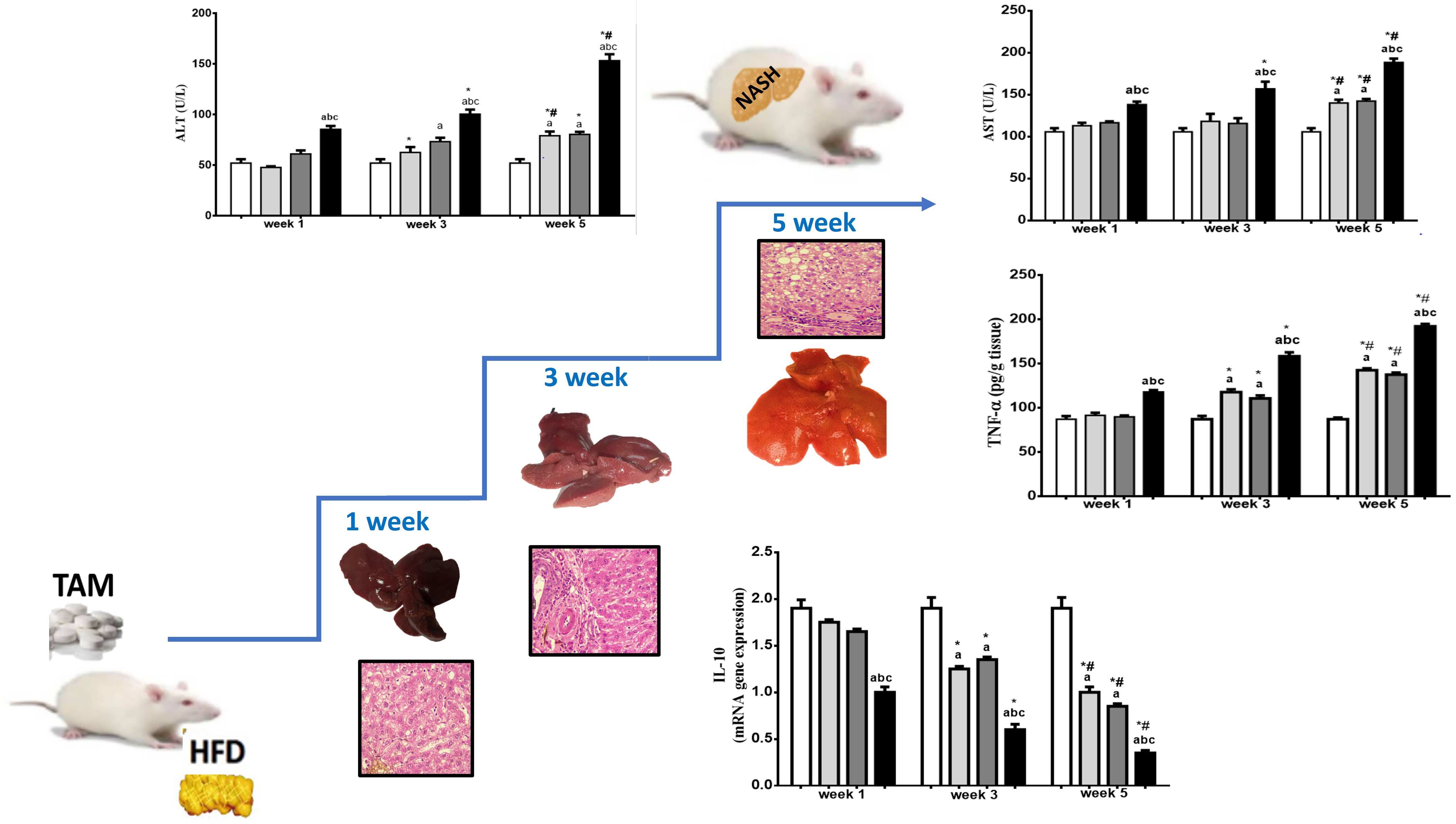

Supplement: Supplementary file 2 — Supplementary Material 2 [file 210_2024_3192_MOESM2_ESM.jpg]
